# The relationship between social and psychological factors with cognitive impairment after stroke: a prospective study

**DOI:** 10.3389/fpsyt.2024.1403027

**Published:** 2024-06-26

**Authors:** Yao Li, Aijie Tang, Lili Ge, Lin Zhang, Ling Chen, Yuhua Xu, Li Wang, Xiaoping Zhu, Qian Wu

**Affiliations:** ^1^ Department of Nursing, Shanghai Tenth People’s Hospital, School of Medicine, Tongji University, Shanghai, China; ^2^ School of Nursing and Health, Henan University, Henan, Kaifeng, China; ^3^ Department of Nursing, Shanghai Ninth People’s Hospital, Shanghai Jiao Tong University School of Medicine, Shanghai, China; ^4^ College of Medicine, Tongji University, Shanghai, China; ^5^ Department of Nursing, Shanghai Ruijin Rehabilitation Hospital, Shanghai, China; ^6^ Department of Intervention, Shanghai Ninth People’s Hospital, Shanghai Jiao Tong University School of Medicine, Shanghai, China

**Keywords:** acute ischemic stroke, post-stroke cognitive impairment, self-perceived burden, depression, social support

## Abstract

**Objectives:**

To investigate the association between social and psychological factors and the risk of cognitive impairment following acute ischemic stroke.

**Materials and methods:**

A prospective study was conducted at Shanghai Tenth People’s Hospital from June 2021 to July 2022. The study focused on social and psychological factors, which were assessed using the Social Support Rating Scale (SSRS), Self-Perceived Burden Scale (SPBS), and Hamilton Depression Scale (HAMD) within 3 days after admission to the hospital. Cognitive function was evaluated using the Montreal Cognitive Assessment at 3 months post-stroke. Logistic hierarchical regression models were used to examine the association between these three indicators and cognitive impairment following a stroke.

**Results:**

Among these patients, cognitive function was assessed in 211 cases at the 3-month follow-up after the initial stroke event. At 3 months post-stroke, 118(55.9%) of the participants experienced cognitive impairment, while 93(44.1%) did not. The scores on the SPBS and HAMD showed significant associations with cognitive impairment at 3 months after stroke. The scores of SPBS [scores: 30~39 vs.<20 points, odds ratio (OR)=2.993 (1.135–7.896); scores: ≥40 vs.<20points, OR=7.382 (1.117–48.799); *P*=0.043] and the HAMD [scores: >7 vs.≤7 points, OR=3.287(1.362~7.936); *P*=0.008]. There were no significant associations observed between SSRS and PSCI.

**Conclusion:**

Early screening for depressive symptoms and focusing on self-perceived burden can be beneficial for decision support for clinicians and improve cognitive function recovery at the 3-month mark post-stroke.

## 1 Introduction

Stroke remains a leading cause of disability in China and around the world (1). Post-stroke cognitive impairment (PSCI) is one of the major sources of morbidity and mortality following acute ischemic stroke (AIS) worldwide, with a prevalence rate ranging from 20% to 82% (2, 3). PSCI is defined as deficits in one or more areas of attention, memory, orientation, language, visuospatial ability, and abstract reasoning occurring within 3 to 6 months after AIS (4). According to a 2-year follow-up study, the incidence of PSCI was highest at 3 months post-stroke (5). Thus, it is of great importance to explore the influence of cognitive function at this time.

About 1/3 of stroke patients will experience PSCI, which seriously affects their quality of life (QoL) and survival time and may lead cause of acquired disability (6). Primary prediction of PSCI is essential for preventing disability, which is important for improving clinical outcomes for patients with PSCI. Previous research indicates that risk factors for PSCI include both physiological, psychological, and social factors, etc. (7–9). Stroke not only has high recurrence and disability rates but also more than 90% of stroke survivors suffer from self-perceived burden, which can occur in both the acute phase (within 4 days post-stroke) and the rehabilitation phase (within 3 months post-stroke) (10, 11). Meanwhile, depression is a serious and common interrelated complication of stroke, especially occurring at the initial stage after the acute event (12). Depression does not just impact mental health but is also associated with increased social isolation (13). Results from an existing study indicate that social support can predict the outcomes of patients, patients with better social support were expected to have a lower risk of depression (14). However, the association between social support and depression may differ among stroke patients compared to other populations.

However, there is a lack of empirical evidence exploring the relationship between psychosocial factors with PSCI at 3 months post-stroke. To further improve the prognosis of clinical stroke patients, it is essential to conduct prospective studies to investigate the level of psychosocial factors and explore the relationship between these factors and PSCI at 3 months post-stroke. Among the psychosocial factors, depression, self-perceived burden, and social support have an important impact on the prognosis of patients with post-stroke cognitive impairment. Therefore, the purpose of our study is to examine the association between early depressive symptoms, self-perceived burden, and the level of social support on the cognitive function of patients with AIS at 3 months post-stroke, informing clinical practice and assist healthcare professionals in treatment decisions.

## 2 Materials and methods

### 2.1 Study design and participants

Our study was conducted at the Shanghai Tenth People’s Hospital affiliated with Tongji University. From a cohort study running from June 2021 to July 2022. Patients were included if they (1) first diagnosis was AIS; (2) patients capable of reading and comprehending adequately to complete the questionnaires; (3) had an understanding of the purpose and process of the study and gave informed consent. Patients were excluded if they (1) with intellectual and psychiatric disorders (e.g., dementia, schizophrenia); (2) with other major diseases (e.g., cancer, severe liver and kidney dysfunction); (3) were taking prescribed anticholinesterase inhibitors or antidepressant medication. The diagnosis of AIS and medications that patients were taking were abstracted from medical records.

Our study was conducted in accordance with the guidelines of the Declaration of Helsinki and approved by the ethics committee of Shanghai Tenth People’s Hospital affiliated with Tongji University (SHSY-IEC-KY-4.0/17–47/01). Written informed consent was obtained from all participants before the study commenced.

### 2.2 Procedure

All participants underwent screening using a structured face-to-face questionnaire to collect demographic information. The Social Support Rating Scale (SSRS), Self-Perceived Burden Scale (SPBS), and Hamilton Depression Scale (HAMD) were measured at baseline (within 3 days of admission); post-stroke cognitive function was measured at the 3-month follow-up examination. Before the assessment, all participants completed the Personal General Condition Questionnaire and were screened by two professional psychiatrists using the HAMD, SSRS, and SPBS scales. At 3 months post-stroke, the study participants or their caregivers were contacted by telephone or text message and invited to the hospital for cognitive function assessments. The investigations were conducted by two trained researchers (YL and AT) who received specialized training for the project and had expertise in stroke care. All completed questionnaires were immediately collected onsite and checked for missing information to ensure data integrity.

### 2.3 Measurements

Demographic and clinical data obtained through a structured face-to-face questionnaire included age, sex, education, marital status, body mass index, possession of medical insurance, smoking and drinking habits, comorbidities (hypertension, diabetes mellitus, atrial fibrillation), stroke severity (15), physical disability (16–18), and stroke history.

Data on social-related factors included the degree of social support, measured by the SSRS. SSRS scores of 45~66 were categorized as high, 23~44 as moderate, and < 22 as low support (19). Psychological factors data included the SPBS and HAMD scales. The SPBS consists of 10 items with three subscales, and the total score ranges from 10~50 points (10). SPBS scores are categorized into four levels: none, mild, moderate, and severe, corresponding to SPBS scores <20, 20≤SPBS score <30, 30≤SPBS score <40, and SPBS score≥40 (20). HAMD scores range from 0~7 points are considered normal, while scores exceeding 7 indicate depressive symptoms, offering a general evaluation of the situation during the latest week (21). Cognitive function data were evaluated using the Montreal Cognitive Assessment (MoCA). The MoCA total score ranges from 0~30 points, with scores ≥26 indicating no cognitive impairment (NCI) and scores <26 indicating cognitive impairment (CI) (22, 23). For participants with 12 years or fewer of education, 1 point was added to their MoCA total score (if <30) (24).

### 2.4 Statistical analysis

Data analysis was carried out using IBM SPSS Statistics version 23.0 (IBM, Armonk, NY, USA). For baseline characteristics, continuous variables are expressed as the means ± standard deviations (M ± SD), and categorical variables are summarized as absolute numbers and percentages. Baseline characteristics between the two groups of participants with and without PSCI were compared using Pearson’s chi-square test. Non-normally distributed data for categorical variables can be analyzed using the Wilcoxon rank-sum test, while continuous variables can be analyzed using the Mann–Whitney U test. Boxplots were utilized to illustrate the distribution of SSRS and SPBS scores based on cognitive impairment (CI/NCI) as measured by MoCA. Logistic hierarchical multiple regression analysis was conducted with three models of variables, including psychosocial indicators (SSRS, SPBS, and HAMD), sociodemographic indicators (age, education level), physical disability and stroke severity indicators. Two-sided P-values < 0.05 were considered statistically significant.

## 3 Results

### 3.1 Patient characteristics

**Figure 1:**
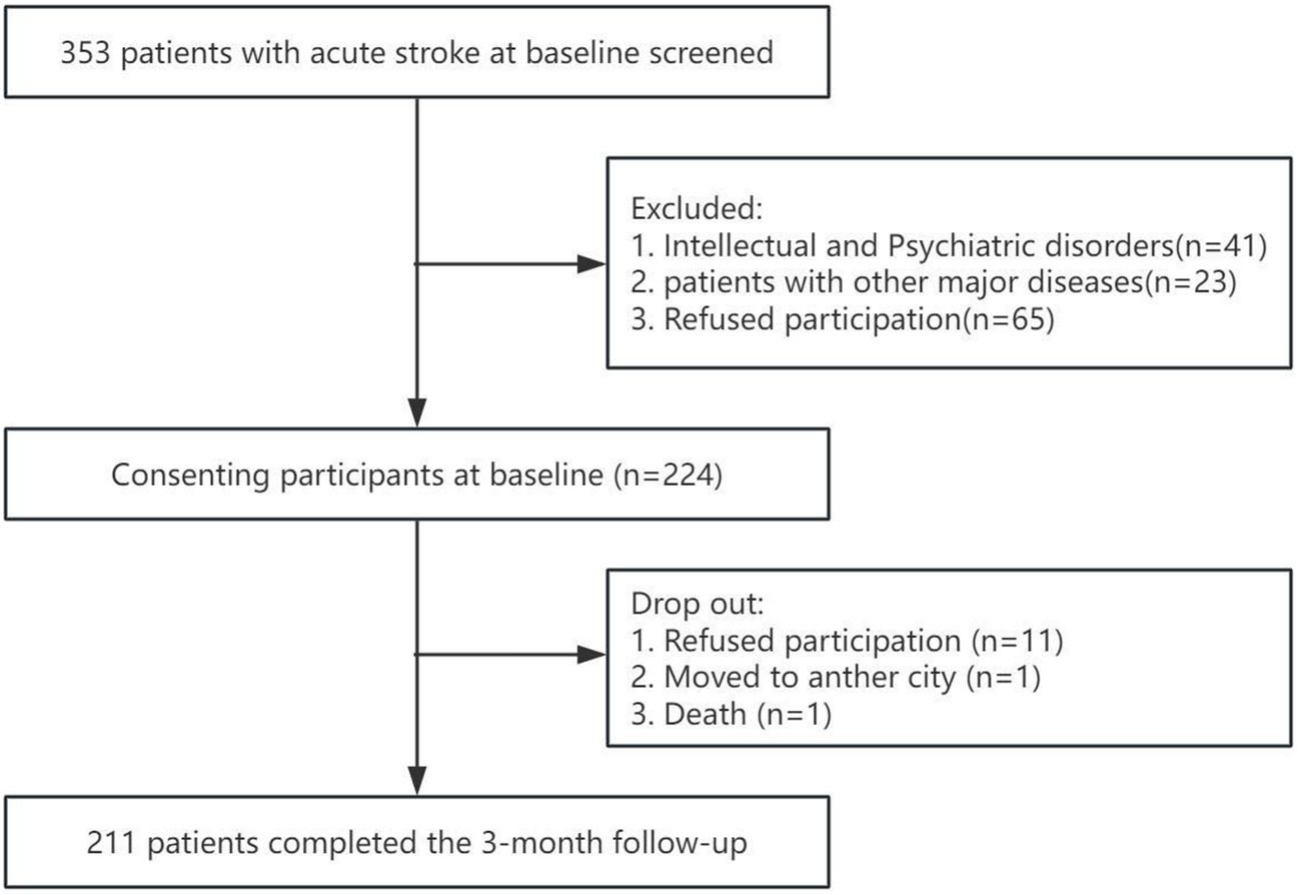
Flowchart of recruitment of patients with stroke.

Initially, a total of 353 patients with AIS for the first time, among whom 224 patients met the inclusion and exclusion criteria. After 3 months follow-up, 13 patients were lost to follow-up, with a 5.8% loss rate. More details of the inclusion process of the study population are provided in **Figure 1**.

**Table 1** summarizes the demographic and clinical characteristics of the 224 enrolled ischemic stroke survivors who enrolled at baseline. The age of patients with AIS ranges from 31 to 88 years. Of the total sample, 67 individuals were female (29.9%), 201 were married (89.7%), and 204 had medical insurance (91.1%). Based on the presence of cognitive impairment three months post-stroke, participants were categorized into two groups: the CI group (n=118) and the NCI group (n=93). The incidence rate of PSCI at 3 months was 55.9%. **Table 2** illustrates significant differences in age (*P* < 0.001), education level (*P* = 0.002), NIHSS (*P* < 0.001), MBI (*P* < 0.001), mRS (*P* < 0.001), SSRS (*P <*0.005), SPBS (*P* < 0.001), and HAMD (*P* < 0.001) scores between the CI and NCI groups.

**Table 1:** Patient characteristics (n= 224).

Characteristics	Total Sample(M ± SD/%)
Age, median (IQR), y	64.0(10.8)
Gender	
Female	67(29.9)
Male	157(70.1)
Marriage	
Married	201(89.7)
Unmarried	23(11.3)
Education	
Primary school education or illiterate	24(10.7)
Junior high school degree	85(37.9)
High school degree	73(32.6)
College degree or above	42(18.8)
BMI, median (IQR), kg/m^2^	23.9(14.0)
Medical insurance	
Having medical insurance	204(91.1)
Self-supporting	20(8.9)
Smoking	
Yes	111(49.6)
No	113(50.4)
Alcohol drinking	
Yes	77(34.4)
No	147(65.6)
Hypertension	162(72.3)
Diabetes mellitus	86(38.4)
Atrial fibrillation	14(6.3)
History of stroke	33(14.7)
Cerebral infarction site	
Vertebrobasilar region	88(39.3)
Bilateral cerebral hemisphere	24(10.7)
Right cerebral hemisphere	44(19.6)
Left cerebral hemisphere	50(22.4)
Watershed infarcts	18(8.0)
NIHSS, median (IQR), scores	3.0(4.0)
MBI, median (IQR), scores	65.0(30.0)
mRS, median (IQR), scores	3.0(2.0)
Total SSRS, mean (standard deviation), scores	36.0(13.0)
Objective support, median (IQR), scores	8.0(4.0)
Subjective support, median (IQR), scores	20.0(8.0)
Degree of social support utilization, median (IQR), scores	7.0(6.0)
Total SPBS, median (IQR), scores	
Physical burden	6.0(5.0)
Emotional burden	11.0(8.0)
Economic burden	4.0(4.0)
HAMD, scores	
≤7	174(77.7)
>7	50(22.3)

BMI, Body Mass Index; NIHSS, National Institutes of Health Stroke Scale; mRS, modified Rankin Scale; MBI, modified Barthel Index.

**Table 2:** Associations between baseline characteristics and post-stroke 3 months cognitive impairment.

Variables	Post-stroke 3 months			
	PSCI (*n*=118)	No-PSCI (*n*=93)	χ^2^/Z	*p* value
Age, median (IQR), y	66.0 (11.3)	62.00 (11.0)	-4.224	<0.001‡
Gender				
Female	36 (30.5)	28 (30.1)	0.004	0.950
Male	82 (69.5)	65 (69.9)		
Marriage				
Married	106 (89.8)	84 (90.3)	0.014	0.906
Unmarried	12 (10.2)	9 (9.7)		
Education				
Elementary school and below	18 (15.3)	5 (5.4)	14.900	0.002‡
Junior high school	48 (40.7)	34 (36.6)		
High school	38 (32.2)	25 (26.9)		
College degree and above	14 (11.9)	29 (31.2)		
Hypertension	85 (72.0)	60 (64.5)	1.367	0.242
Diabetes mellitus	53 (44.9)	30 (32.3)	3.492	0.062
Atrial fibrillation	8 (6.8)	6 (6.5)	0.009	0.924
History of stroke	20 (16.9)	8 (8.6)	3.148	0.076
NIHSS, scores	4.0 (5.0)	3.0 (3.0)	-4.099	<0.001‡
MBI, scores	60.0 (25.0)	75.0 (30.0)	-5.743	<0.001‡
mRS, scores	3.0 (2.0)	3.0 (2.0)	-5.164	<0.001‡
SSRS, scores			7.703	0.021‡
<22	8 (6.8)	1 (1.1)		
23–44	95 (80.5)	70 (70.5)		
≥45	15 (12.7)	22 (23.7)		
SPBS, scores			20.376	<0.001‡
<20	32 (27.1)	51 (54.8)		
20–29	45 (38.1)	29 (31.2)		
30–39	29 (24.6)	11 (11.8)		
≥40	12 (10.2)	2 (2.2)		
HAMD, scores			13.618	<0.001‡
≤7	80 (67.8)	83 (89.2)		
>7	38 (32.2)	10 (10.8)		

‡ Statistically significant (P < 0.05).

NIHSS, National Institutes of Health Stroke Scale; mRS, modified Rankin Scale; MBI, modified Barthel Index.

### 3.2 SSRS, SPBS for patients with PSCI

**Figure 2:**
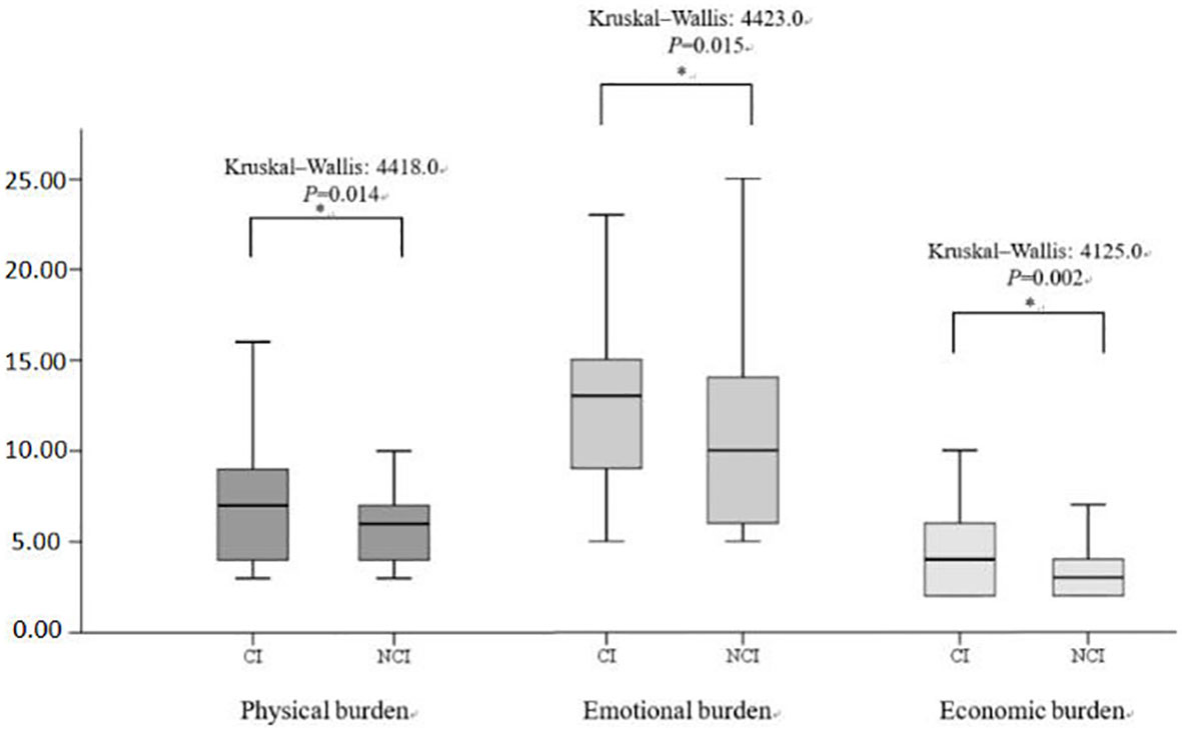
Distribution of the three SSRS subscales and cognition (cognitive impairment/normal). "*" represents P < 0.005.

**Figure 3:**
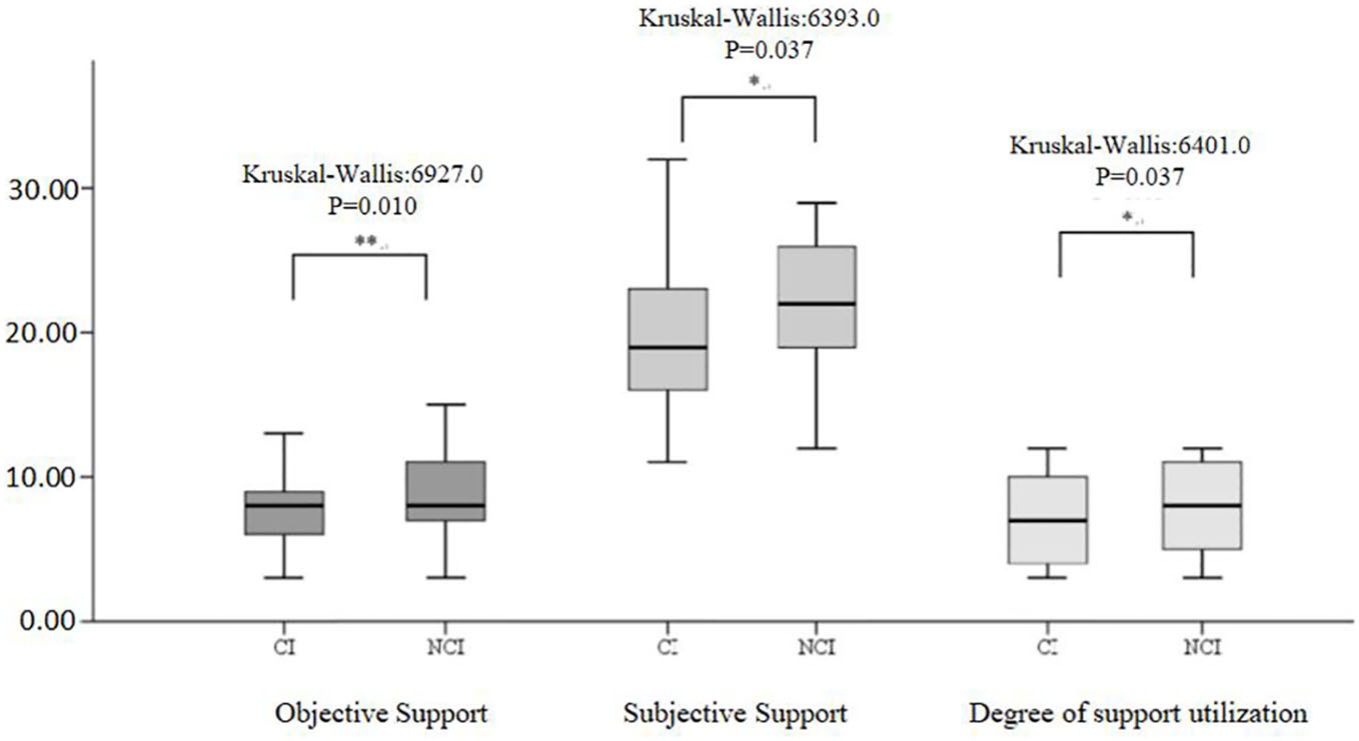
Distribution of the three SPBS subscales and cognition (cognitive impairment/normal). "*" represents P > 0.005, "**" represents P ≤0.001.

**Figure 4:**
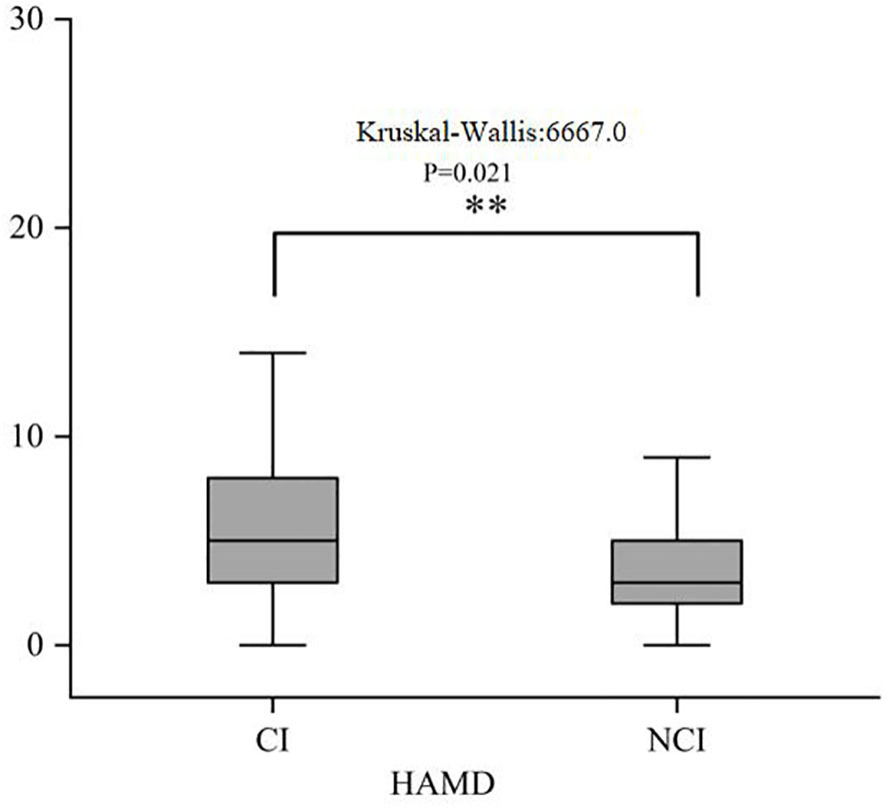
Distribution of the HAMD. "**" represents P ≤0.001.

**Figure 2** illustrates the distribution of the three subscales in SSRS (objective support, subjective support, and degree of social support utilization) scales for the CI and NCI groups. The Kruskal–Wallis values were 6927.0, 6393.0, and 6401.0 for objective support, subjective support, and the degree of social support utilization, respectively. Significant differences (*P* < 0.05) were observed in the median SSRS scores between the groups at 3 months post-stroke. **Figure 3** illustrates the distribution of the three subscales in SPBS (physical burden, emotional burden, and economic burden). The Kruskal–Wallis values were 4418.0, 4423.0, and 4125.0 for physical burden, emotional burden, and economic burden, respectively. Significant differences (*P* < 0.05) were observed in the median SPBS scores between the groups at 3 months post-stroke. **Figure 4** illustrates the distribution of the HAMD. The Kruskal–Wallis values were 6667.0 for HAMD, respectively. Significant differences (P < 0.05) were observed in the median HAMD at 3 months post-stroke.

### 3.3 Association between SSRS, SPBS, and HAMD on CI

**Figure 5:**
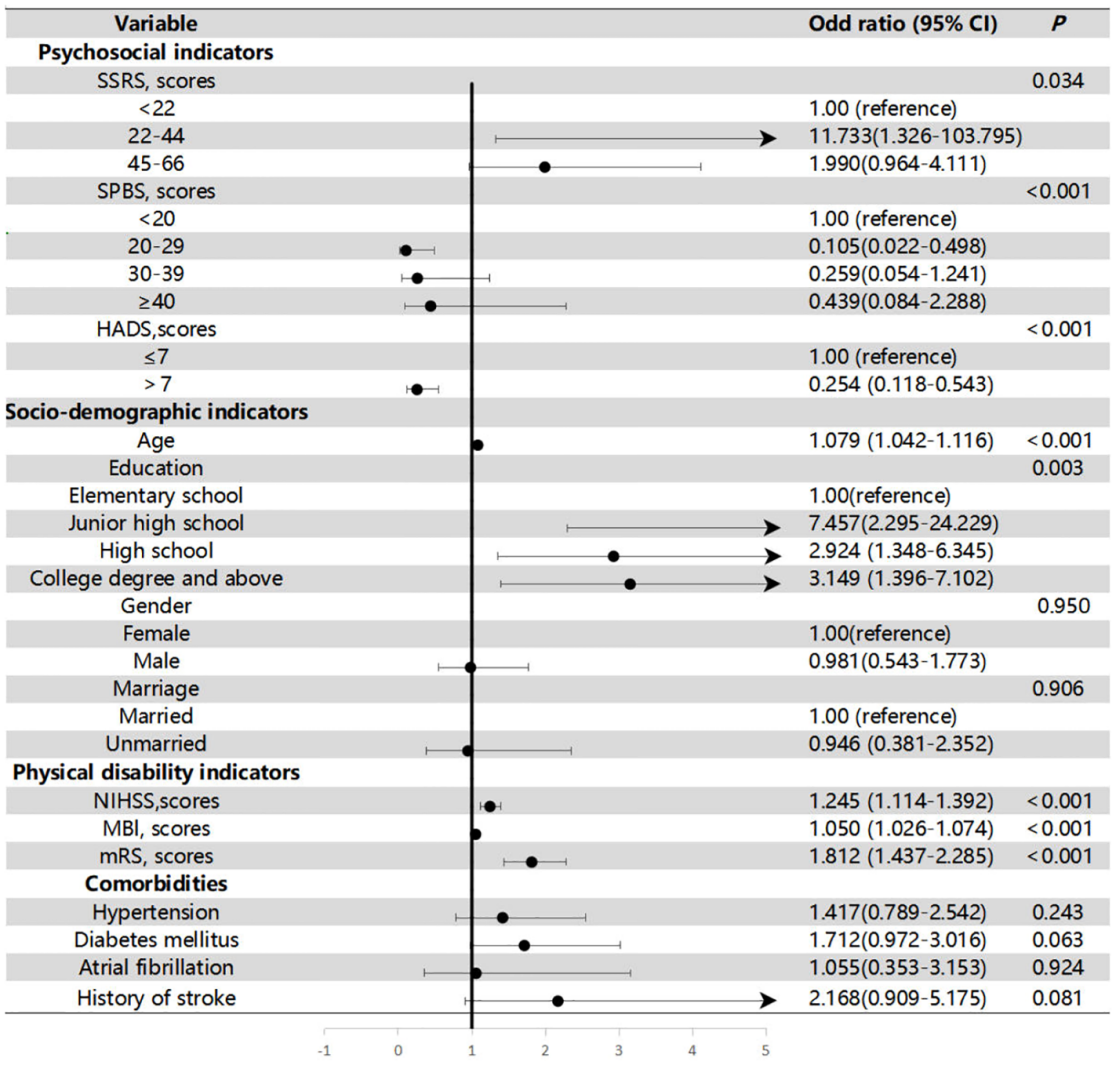
Relationship between all variables and cognition at 3 months post-stroke.

We used cognitive function as the dependent variable three months later and the variables that showed statistical significance in the univariate analysis as the independent variables to conduct regression analysis on the factors influencing CI at 3 months post-stroke. **Figure 5** illustrates the association between all variables and cognition at 3 months post-stroke. Age [OR=1.079, 95% CI 1.042–1.116, *P*<0.001] and education level [junior high school vs. elementary school, OR=7.457, 95% CI 2.295–24.229; high school vs. elementary school, OR=2.924, 95% CI 1.348–6.345; college degree and above vs. elementary school, OR=3.149, 95% CI 1.396–7.102; all *P*_s_=0.003] were significantly and independently associated with cognition at 3 months post-stroke. The VIF for all factors was less than 10(1.000–8.515), indicating no multicollinearity among variables in the multivariate model.

Furthermore, **Table 3** illustrates the association between the variables of interest and cognitive function after the inclusion of variables in three models. Model 1 included the SSRS, SPBS, and HAMD scores, which were significantly associated with cognition at 3 months post-stroke. Model 2 included age and education level, while Model 3 included NIHSS, MBI, mRS to adjust for group differences. The associations remained highly significant for PSCI with SPBS [scores:30–39 vs. <20 points, odds ratio (OR)=2.993(1.135–7.896); scores:≥40 vs. <20 points, OR=7.382 (1.117–48.799); *P*=0.043], HAMD [scores:>7vs.≤7 points, OR=3.287 (1.362–7.936); *P*=0.008].

**Table 3:** Results of hierarchical multiple regression analysis predicting cognitive impairment at three months post- stroke.

Predictors	Model l		Model 2^a^		Model 3^b^	
	Odds ratio (95% CI)	P Value for Trend	Odds ratio (95% CI)	P Value for Trend	Odds ratio (95% CI)	P Value for Trend
**SSRS, scores**		0.037		0.036		0.104
<22	Reference		Reference		Reference	
23-44	0.170(0.021-1.388)		0.179(0.021-1.543)		0.207(0.021-2.046)	
≥45	0.085(0.010-0.754)		0.083(0.009-0.782)		0.103(0.009-1.149)	
**SPBS, scores**		<0.001		<0.001		0.043
<20	Reference		Reference		Reference	
20-29	2.473(1.300-4.703)		2.419(1.213-4.822)		1.863(0.879-3.948)	
30-39	4.202(1.845-9.567)		2.375(2.218-13.305)		2.993(1.135-7.896)	
≥40	9.562(2.008-45.544)		9.448(1.714-52.072)		7.382(1.117-48.799)	
**HAMD, scores**		<0.001		0.001		0.008
≤7	Reference		Reference		Reference	
>7	3.942(1.841-8.441)		3.966(1.777-8.850)		3.287(1.362-7.936)	

^a^Adjusted for age, education.

^b^Adjusted for NIHSS, MBI, mRS, age, education.

## 4 Discussion

This study focused on assessing social support, self-perceived burden, and depression in patients with AIS from a psychosocial viewpoint, examining the correlation of these variables with cognitive impairment at 3 months post-stroke. Our results showed a 55.9% incidence of PSCI at 3 months post-stroke, similar to the results of previously published studies (25, 26). Thus, it is of great importance to explore the influence of cognitive function at this time point. We found that patients with high self-perceived burden and severe depression contributed significantly to predicting the likelihood of cognitive dysfunction in AIS survivors at the 3-month post-stroke mark. This finding suggests that psychological intervention in advance may be an important factor in protecting cognitive function among AIS survivors.

Currently, with the development of social medicine and health, mental health has garnered wide attention in healthcare discussions (27). It has become an important indicator of the quality of care for patients with various illnesses in both Western and Eastern cultures. However, the clinical focus on stroke in China is still primarily on disease management, often neglecting mental health and cognitive function (28). A multi-center study revealed that 62.7% of stroke survivors necessitated psychological support, and underscored the insufficiency of available psychological support services (29). Additionally, while health insurance now covers over 95% of Chinese citizens (30), there is still a lack of resources to address mental health needs, which is attributed to constraints in medical resources. The limited availability of rehabilitation services indicates the neglect of aspects crucial for maximizing recovery in speech, cognitive, and emotional function recovery in China, despite the significant demand for psychological support among Chinese stroke patients. Our findings contribute to enhancing the PSCI risk assessment system in China and emphasize the significance of healthcare providers prioritizing addressing depression and the self-perceived burden among post-stroke patients.

Our study showed that education level and age play important roles in the occurrence of cognitive impairment in stroke patients at the 3-month, which may be caused by different cognitive reserve and brain plasticity. Before the age of 20, brain plasticity reaches its maximum, and acquired cognitive reserve is positively related to educational level (31). The results of a large cohort study show that higher education is associated with a more rapid recovery of cognitive function during the first 3 months post-stroke because high levels of education might have had more synapses, or more robust brain networks that effectively resisted brain damage (32, 33). Meanwhile, with the increase of age, brain function itself is declining, and young people’s plastic repair ability is high and they recover quickly. Moreover, hypertension, diabetes, and other comorbidities did not exhibit statistical significance in this study, possibly influenced by variations in cognitive impairment diagnostic criteria and the characteristics of the study cohorts. Further large-sample multicenter research is needed in the future to verify the relationship between them.

Moreover, our study reports a novel and significant finding that a higher self-perceived burden is a predictor of PSCI at the 3-month follow-up. One explanation for this might be that at 3 months post-stroke, the majority of survivors remained phenotypic on physical burden includes impairment of executive function, visuospatial ability, and instrumental activities of daily living post-stroke. These physical burdens may directly contribute to PSCI (34). Additionally, cognitive function post-stroke can be indirectly impacted by patients with a heavy emotional burden, such as negative psychological experiences (10). While stroke patients undergoing rehabilitation often exhibit enhanced functional outcomes, those with high self-perceived burdens are typically less optimistic (35). The research results of Wei et al. are similar to our views (11). Therefore, it is crucial to prioritize assessing the self-perceived burden of stroke patients and implement timely interventions to enhance their cognitive function.

Our study further illustrated the association between early depressive symptoms and cognitive function at the 3-month post-stroke. This finding contributes to the current literature by indicating that early depression may serve as a predictor of cognitive impairment three months post-stroke. Williams et al. (7) confirmed that post-stroke depression (PSD) correlated with PSCI in both general and specific cognitive domains. Indeed, it has also been shown that impaired cognition at the acute phase could not predict PSD 3 months later, but it is still significant in the female subgroup (9). Similar to our study, Terroni et al. (36) also confirmed that early depressive symptoms were associated with cognitive impairment during the first 3 months post-stroke, especially executive dysfunction. To date, the relationship between PSD and PSCI is interactive, and causality between the two is uncertain; further studies should investigate this. Our study highlights the importance of early screening for depressive symptoms in patients, which can facilitate early intervention to prevent PSCI adverse outcomes during the later stages of recovery.

The results of the present study revealed the important impact of social support on PSCI in patients with AIS. Our study shows that due to equal medical care support during hospitalization, about 96% of stroke patients report that they can obtain social support, but 58.4% of patients will still suffer from PSCI. The reason for this phenomenon may be that severe depression will occur under the premise of social support. Symptoms or self-perceived burden still increase the risk of 3-month PSCI.

Consistent with the findings of Salinas et al. (37) and Saeed’s study (38), we did not find that social support did not impact cognitive impairment three months post-stroke. However, the existing evidence on the relationship between social support and PSCI is conflicting (14, 37–39), which is probably due to significant heterogeneity in the definition and evaluation of social support. Several previous studies have shown that the degree of social support for stroke patients has a significant impact on the outcomes of stroke patients (14, 39). Maybe higher levels of pre-stroke social support reflect poorer pre-stroke function (40). Future longitudinal studies are still warranted to further explore SSRS differences in PSCI prevalence and incidence.

The study has several limitations. Firstly, the selection of stroke patients from a single center may have limited the generalizability of the result. Secondly, there was no follow-up on PSCI over various time periods in this study. Thirdly, since all the patients we included were admitted to the hospital for the first time with acute ischemic stroke, we were unable to perform MoCA measurements at baseline. Furthermore, cognitive impairment was not graded and only presence and absence were mentioned. The authors plan to conduct a multi-center, multi-ethnic, longer follow-up cycle study in the next phase to achieve a more representative sample group design and confirm the causal relationship among depressive symptoms, self-perceived burden, social support, and 3-month PSCI.

## 5 Conclusions

In summary, our results provide evidence that self-burden and depression are associated with an increased risk of incident PSCI. Screening for depressive symptoms and focusing on self-perceived burden is recommended in clinical practice to enhance the cognitive function of AIS patients at the three-month post-stroke stage. Attention should be paid to the care of depressive and higher self-burden AIS patients, and effective preventions, should be implemented to reduce the prevalence of 3-month PSCI among AIS patients.

## Data availability statement

The original contributions presented in the study are included in the article/supplementary material. Further inquiries can be directed to the corresponding authors.

## Ethics statement

The studies involving humans were approved by the Shanghai Tenth People’s Hospital Ethics Committee. The studies were conducted in accordance with the local legislation and institutional requirements. The participants provided their written informed consent to participate in this study. Written informed consent was obtained from the individual(s) for the publication of any potentially identifiable images or data included in this article.

## Author contributions

YL: Methodology, Software, Validation, Visualization, Writing – original draft, Writing – review & editing. AT: Data curation, Investigation, Methodology, Visualization, Writing – original draft. LG: Methodology, Visualization, Writing – original draft. LZ: Investigation, Visualization, Writing – original draft. LC: Formal analysis, Investigation, Visualization, Writing – original draft. YX: Visualization, Writing – original draft, Investigation. LW: Methodology, Investigation, Writing – review & editing. XZ: Formal analysis, Visualization, Writing – original draft. QW: Formal analysis, Funding acquisition, Project administration, Supervision, Visualization, Writing – original draft, Writing – review & editing.
